# Significant Increase in Depression in Women With Primary Dysmenorrhea: A Systematic Review and Cumulative Analysis

**DOI:** 10.3389/fpsyt.2021.686514

**Published:** 2021-08-05

**Authors:** Shankun Zhao, Weizhou Wu, Ran Kang, Xiaolan Wang

**Affiliations:** ^1^Department of Urology, Taizhou Central Hospital, Taizhou University Hospital, Taizhou, China; ^2^Department of Urology, Maoming People's Hospital, Maoming, China; ^3^Department of Urology, The First Affiliated Hospital of University of South China, Hengyang, China; ^4^Reproductive Center of Medicine, The First Affiliated Hospital of University of South China, Hengyang, China

**Keywords:** primary dysmenorrhea, depression, systematic review, cumulative analysis, risk

## Abstract

Women with primary dysmenorrhea are vulnerable to develop a depressive disorder, which is a common form of psycho-disturbance. However, clinical findings are inconsistent across studies, and the evidence has not been previously synthesized. This study aims to investigate whether primary dysmenorrhea is associated with a higher risk of depression via a cumulative analysis. Four electronic databases were systematically searched for the eligible studies. The combined effect was assessed by analyzing the relative risk (RR) and standard mean differences (SMD) with a 95% confidence interval (CI). This cumulative analysis was registered on the PROSPERO (ID: CRD42020169601). Of 972 publications, a total of 10 studies involving 4,691 participants were included. Pooled results from six included studies showed that primary dysmenorrhea was associated with a significant depressive disorder (*RR* = 1.72, 95%CI: 1.44 to 2.0, *P* < 0.001; heterogeneity: *I*^2^ = 0%, *P* = 0.544). In addition, synthesis results from two studies provided the BDI scores suggested that dysmenorrhea had significantly higher scores when compared to non-dysmenorrhea (*SMD* = 0.47, 95% CI: 0.31–0.62, *P* < 0.001; heterogeneity: *I*^2^ = 0%, *P* = 0.518). However, in the two studies providing the PROMIS T-Score, the pooled result showed that there was no significant difference between women with dysmenorrhea and those without dysmenorrhea (*P* = 0.466). The overall quality of the evidence in our study was judged to MODERATE. The present study has confirmed the positive relationship between primary dysmenorrhea and depression. Social supports and medical help from pain management physicians or psychologists are important interventions for women with dysmenorrhea-suffering depressive disorder.

## Introduction

Primary dysmenorrhea, a common gynecologic problem among women of reproductive age, is characterized by painful uterine cramps that occur before or during menstruation (1, 2). Women with dysmenorrhea are usually felt pain in the lower part of the abdomen without any pelvic diseases (i.e., endometriosis, pelvic inflammatory disease, uterine leiomyoma, or intrauterine contraceptive device) (3). Dysmenorrhea has even been considered a genuine chronic pain condition, occurring on a regular basis for most women. The primary cause of dysmenorrhea is thought to be correlated with prostaglandins, the hormones that induce muscle contractions and reduce blood flow and oxygen to the uterus (4). The prevalence of dysmenorrhea in young women varies from different countries or regions, ranging from 16 to 95% depending on the method of assessment (1, 5), which is severe in 2–29.8% of cases (6). Though dysmenorrhea is not life-threatening, it is one of the important factors that lower the quality of life, reduce social activities, and absent from school or work among young women. And surprisingly, dysmenorrhea has received slight scientific and clinical attention. Based on relevant studies investigating the different impacts on life in women with or without dysmenorrhea, women with dysmenorrhea are vulnerable to have higher levels of depression, anxiety, somatization, negative self-perception, and hostility (7). Besides, these women are also prone to reduce productivity, creativity, and job performance (8). Among these complications, depressive disorder is one of the most commonly reported issues in women with dysmenorrhea.

Mounting evidence has emerged suggesting that mood dysfunction was comorbid with dysmenorrhea condition (9). According to the previous studies, there is a positive association between primary dysmenorrhea and depressive disorder, and these two factors often cause a vicious circle of symptoms. It was suggested that depression might serve as a risk factor for dysmenorrhea (10). Tavallaee et al. (11) reported that women with higher depression tended to have more severe dysmenorrhea. Oppositely, some researchers showed that young women with dysmenorrhea have a higher risk of developing depression (12). A study reported by Liu et al. (13) indicated that subjects with primary dysmenorrhea were susceptible to depression when compared to those without dysmenorrhea (Self-rating distress scale: 34.36 ± 1.95 vs. 28.8 ± 1.58). In addition, Ambresin et al. (7) demonstrated that adolescent girls with severe dysmenorrhea had a 1.87-fold increased risk of depression as compared with those with no/mild/moderate dysmenorrhea. However, some studies failed to support such an association. In a large sample case-control study, László et al. (14) found that the prevalence of depressive symptoms in women with dysmenorrhea was comparable to the subjects without dysmenorrhea (6.46 vs. 5.0%, *P* = 0.08).

Though a positive association between dysmenorrhea and depression is speculated, the evidence is still controversial, and a directly calculated prevalence of depression is presently lacking. Therefore, the current systematic review and meta-analysis aim to summarize all the evidence focused on this topic and show a quantified result to better determine the level of risk in women with dysmenorrhea when compared to those healthy women without dysmenorrhea. If the synthesis results have confirmed this relationship, it is instructive and meaningful to help the clinicians being conscious of the hazardous effect of dysmenorrhea for developing depression and take some interventions to alleviate dysmenorrhea for the sufferers.

## Methods

The protocol and report of this systematic review and meta-analysis were followed by the Preferred Reporting Items for Systematic Reviews and Meta-Analyses (PRISMA) guidelines. The PRISMA checklist was illustrated in Supplementary Figure 1. Furthermore, we have registered this meta-analysis on the PROSPERO. For more detailed information, please visit the website of PROSPERO to access it (ID: CRD42020169601, http://www.crd.york.ac.uk/PROSPERO).

### Data Sources and Searches

Four electronic databases, such as MEDLINE (PubMed), EMBASE (OVID), the Cochrane Library, and the PsychINFO were systemically retrieved by two authors up to January 07, 2021. The searching strategy applied for identifying the qualified studies in PubMed databases by using the MeSH and the terms was: ((((((((([“Depression”(Mesh)] OR Depressions) OR Depressive Symptoms) OR Depressive Symptom) OR Symptom, Depressive) OR Symptoms, Depressive) OR Emotional Depression) OR Depression, Emotional) OR Depressions, Emotional) OR Emotional Depressions) AND ((((((((([“Dysmenorrhea”(Mesh)] OR Dysmenorrheas) OR Pain, Menstrual) OR Menstrual Pain) OR Menstrual Pains) OR Pains, Menstrual) OR Menstruation, Painful) OR Menstruations, Painful) OR Painful Menstruation) OR Painful Menstruations).

### Measurement of Dysmenorrhea and Depression

Exactly, dysmenorrhea is a problem, rather than a disease, that periodically makes people uncomfortable due to menstrual cramps during the menstrual cycles. Assessments for dysmenorrhea or pain severity include but are not limited to specific questions, the Visual Analogue Scale (VAS), McGill Pain Questionnaire (MPQ), verbal multidimensional scoring system, Numeric Pain Rating Scale, the Wong-Baker FACES Pain Rating Scale, and scale of noncyclic pelvic pain. The definition of depression is qualified following the international classification of diseases (ICD) codes. Depression is an affective disorder manifested by either a dysphoric mood or loss of interest or pleasure in usual activities. Assessments for depression are according to Beck Depression Inventory (BDI), The NIH Patient Reported Outcomes Measurement Information System (PROMIS), the Edinburgh Depression Scale, the Depression Anxiety and Stress Scale (DASS-21), etc.

### Study Selection

To be included in this meta-analysis, epidemiologic studies had to report the association between dysmenorrhea and depression. Only studies reporting with the English language and human participants were included. Those potential studies were reviewed and further assessed whether they met the predefined inclusion criteria. In line with the Patient, Intervention, Comparison, Outcome, and Study design (PICOS) standard, the question that guided the current meta-analysis was: Does dysmenorrhea increase the risk of depression/depressive disorder? The PICOS evidence for this meta-analysis has consisted of the following combinations: patients complained of dysmenorrhea or painful menstruation or menstrual Pain (P); a history of dysmenorrhea (I); compared with the healthy general population who did not suffer dysmenorrhea (C); diagnosed with depression or depressive disorder (O); no limitation on study designs (S). Besides, studies provided the odds ratios (OR), relative risk (RR), and standard mean differences (SMD) with 95% confidence intervals (CI) could be also included. In this study, we excluded the irrelevant studies according to the pre-specified exclusion criteria, including lack of information of the general populations who did not complain of dysmenorrhea; duplicated data or previous publications of the same clinical trials; review studies; case reports; letters; comments; meeting abstracts; and animal experiments. Based on the PICOS evidence and the inclusion criteria, the process of study selection was conducted by two authors independently. Any ambiguities or discrepancies were resolved by the corresponding author.

### Data Extraction

Two authors independently evaluated and extracted the necessary data from each included study by using a data collection form. Corresponding information includes the name of the first author; publication year; country/region of study origin; study design; mean age, total number, and cases of dysmenorrhea from the two groups; age at menarche; menstrual bleeding duration; RR with 95% CI) or SMD with 95% CI; assessment of dysmenorrhea or pain severity; and assessment of depression.

### Quality Assessment

Study quality assessments were also conducted by two investigators independently. Interrater variability among manual scorers was evaluated from the agreement between the two scorers. Quality assessments of the eligible cross-sectional studies were performed using the cross-sectional study quality methodology checklist. This checklist consists of 11 items in which each criterion is assigned a score, and studies with scores of 0–3, 4–7, and 8–11 are rated too low quality, moderate quality, and high quality, respectively. The methodological quality of the case-control studies is evaluated by the Newcastle–Ottawa Scale. This scale contains nine domains and the conformity is assigned with one score. Eligible studies with scores of 0–3, 4–6, and 7–9 were rated as low quality, moderate quality, and high quality, respectively. Furthermore, we employed the grading of recommendations assessment, development, and evaluation (GRADE) approach to exert the absolute estimates of the risk of depression in patients with dysmenorrhea and meanwhile rank the overall quality of evidence.

### Meta-Analyses

Quantifying the association between dysmenorrhea and depression in women is the main objective of the current study. The dichotomous variables were calculated and presented with the RR and its 95% CI, while the continuous variables were calculated according to the SMD and its 95% CI. *P* < 0.05 were considered statistically significant. The heterogeneity test was performed using the *I*^2^ statistic and the Cochrane *Q* statistic (significance level at *P* < 0.10 with *Q* test; *I*^2^ > 50% was rated to substantial heterogeneity). In this meta-analysis, a random-effects model rather than a fixed-effects model was conducted due to a high likelihood of between-study variance for differences in the study design and the sample sizes. Sensitivity analyses were carried out to better detect the potential source of the heterogeneity. Publication bias analyses were conducted using the funnel plot as well as Begg's rank correlation test and Egger's regression asymmetry test. All statistical analyses were conducted via the STATA software (version 13.0, Stata Corp LP, Texas, USA).

## Results

### Literature Search and Eligible Study Characteristic

**Figure 1:**
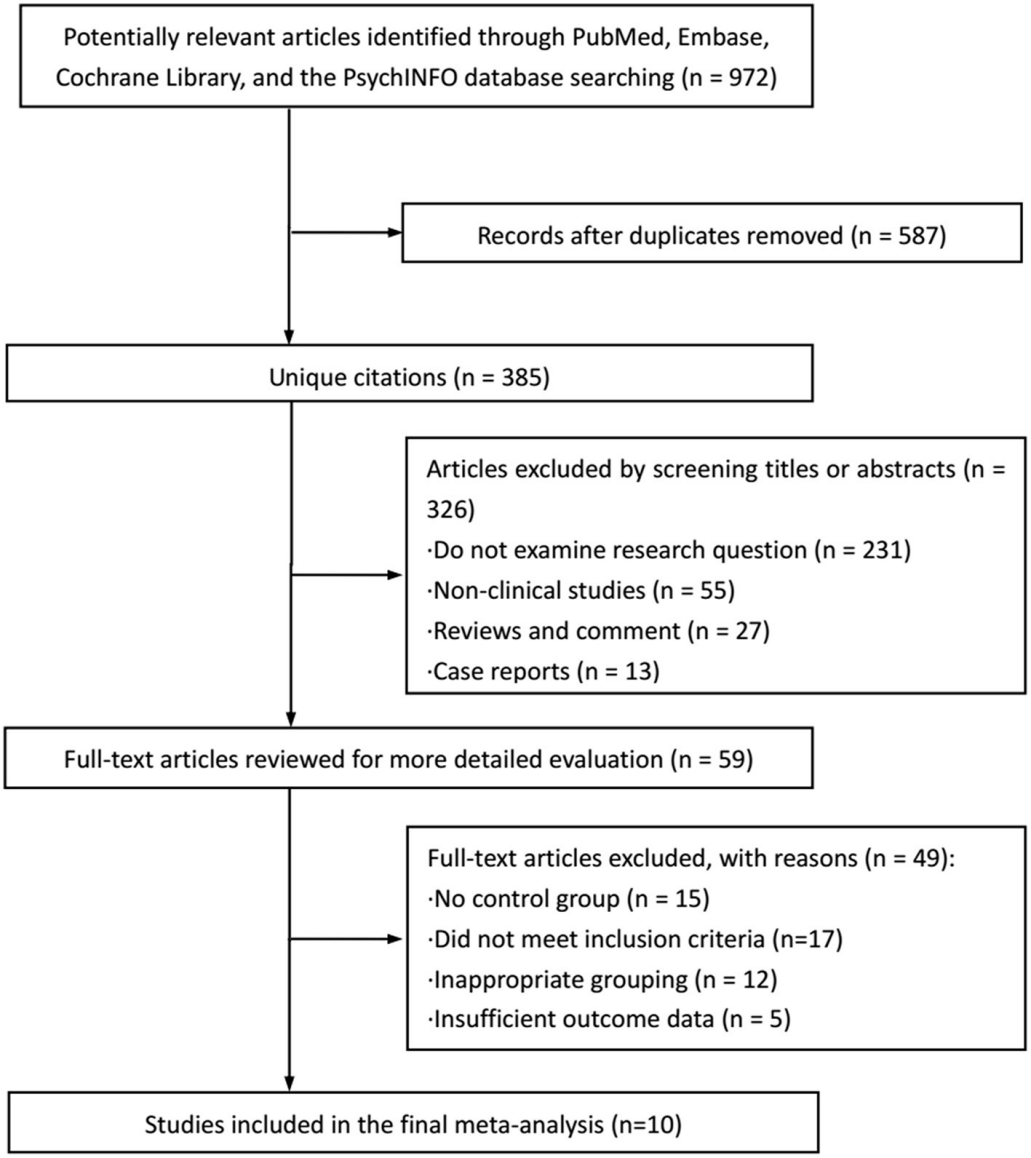
Flow chart of study selection.

The search flowchart for identifying the eligible studies was shown in Figure 1. The initial database search yielded 972 records, of which 376 from PubMed, 248 from Embase, 217 from the Cochrane Library, and 131 from the PsychINFO database. Finally, 10 relevant publications (14–23) contained data that met our predefined inclusion criteria. A total of 4,691 subjects and 2,130 dysmenorrhea cases were involved. Of the 10 included studies evaluated, 6 studies (14–19) provided the dichotomous variables (cases of depression) that could be calculated for the pooled RR via a meta-analysis, while 4 studies (20–23) provided the continuous variables (scores for depression), which were calculated for the pooled SMD. Among the 10 studies included in this meta-analysis, 5 studies (15, 17, 21–23) were cross-sectional designed and the remainder (14, 16, 18–20) were case-control designed. The trial publication years of the 10 selected studies ranged from 2006 to 2019. The mean age in women with dysmenorrhea and women without dysmenorrhea was 18–50 years and 18–43 years, respectively. One trial was conducted in Hungary (14), one in Gambia (15), one in Georgia (16), one in China (19), two in Iran (17, 21), two in Turkey (18–20), and two (22, 23) in the USA. The age at menarche of the participants ranged from 12.4 ± 1.1 years to 13.5 ± 1.2 years. Menstrual bleeding duration ranged from 4.5 ± 1.2 days to 6.4 ± 1.1 days. The characteristics of the 10 eligible studies are summarized in Table 1.

**Table 1:** Characteristics of the included studies.

**Study**	**Study design**	**Mean age (years)**	**Age at menarche**	**Menstrual bleeding duration (days)**	**Study group case/total**	**Control group case/total**	**RR (95% CI) or SMD (95% CI)**	**Assessment of dysmenorrhea or pain severity**	**Assessment of depression**
**The 6 included studies that provided the sufficient data which could be calculated for the pooled RR via a meta-analysis:**									
Coleman (15) 2006 Gambia	Cross-sectional	15-54	NA	NA	14/33	38/210	2.34 (1.44, 3.83)	NA	The Edinburgh Depression Scale
László (14) 2009 Hungary	Case–control	S: 37.2 ± 9.4C: 38.5 ± 9.3	NA	NA	11/165	33/656	1.33 (0.68, 2.57)	Specific question	BDI
Gagua (16)2013 Georgia	Case–control	S: 16.0 ± 1.4C: 15.5 ± 0.9	S: 12.6 ± 1.0 C: 12.7 ± 1.0	S: 4.9 ± 1.4C: 4.5 ± 1.2	49/276	9/148	2.92 (1.48, 5.78)	The VAS and MPQ	BDI
Faramarzi (17) 2014 Iran	Cross-sectional	Both S & C:20.4 ± 1.6	66.4% more than 12 years	Both S & C:6.4 ± 1.1	81/180	43/180	1.88 (1.39, 2.56)	A verbal multidimensional scoring system	The DAS-21
Uçar (18) 2018 Turkey	Case–control	Both S & C:20.9 ± 1.7	Both S & C: 13.5 ± 1.2	76.6% lessthan 6 days	127/471	81/471	1.57 (1.22, 2.01)	Numeric PainRating Scale	BDI
Meng (19) 2019 China	Case–control	29.04 ± 4.29	NA	NA	Calculated by the RR^#^: 50/192	Calculated by the RR^#^: 22/168	Original data from the study: 1.95 (1.24, 3.06)	Specific question	The Edinburgh Postnatal Depression Scale
**The 2 included studies that provided the BDI scoring which were calculated for the pooled SMD via a meta-analysis:**									
Balik (20) 2014 Turkey	Case– control	S: 17.7 ± 1.3C: 17.8 ± 1.1	S: 13.2 ±1.2 C: 13.4 ± 1.1	NA	NA/108 (11.9 ± 10.8)	NA/159(7.1 ± 7.6)	0.53 (0.28, 0.78)	The Wong-Baker FACES Pain Rating Scale	BDI
Bahrami (21) 2017 Iran	Cross- sectional	S: 14.6 ± 1.5C: 14.6 ± 1.7	S: 12.4 ± 1.1 C: 12.6 ± 1.1	80%: 4-7 days	NA/322 (12.0 ± 10.0)	NA/148(8.0 ± 7.8)	0.43 (0.23, 0.62)	Specific scale	BDI
**The 2 included studies that provided the PROMIS T-Score which were calculated for the pooled SMD via a meta-analysis:**									
Hellman (22) 2018 USA	Cross- sectional	S: 24 ± 6C: 24 ± 7	NA	NA	NA/98 (53 ± 1)	NA/35(55 ± 1)	−2.0 (-2.46,−1.54)	Numerical rating scale	PROMIS
Zuckerman (23) 2018 USA	Cross- sectional	S: 34.1 ± 7.6C: 35.2 ± 7.1	NA	S: 5.3 ± 1.5C: 4.8 ± 1.5	NA/285 (53.6 ± 9.2)	NA/386(50.9 ± 8.8)	0.3 (0.15, 0.45)	Scale of noncyclic pelvic pain	PROMIS

*BDI, Beck Depression Inventory; VAS, The Visual Analogue Scale; MPQ, McGill Pain Questionnaire; DAS-21, Depression Anxiety and Stress Scale; PROMIS, The NIH Patient Reported Outcomes Measurement Information System; RR, Relative Risk; SMD, standard mean differences; CI, Confidence Interval; S: Study group, women with dysmenorrhea; C: Control group, women without dysmenorrhea; NA, Not Available; #: Estimated using the methods described in the Cochrane Handbook*.

### Study Quality and Quality of the Evidence

Across the 10 included studies, they were rated to a medium to high methodological quality. The proportion of high-quality studies was 20% (2/10). Among the five cross-sectional studies, four studies (15, 17, 22, 23) were assessed as moderate quality, and the remaining one study (21) was high quality (Supplementary Table 1). In the five case-control studies, four studies (14, 16, 18, 20) were judged to moderate quality, and only one study (19) was evaluated as high quality (Supplementary Table 2).

Table 2 showed the calculated results of the quality of evidence using the GRADE-pro. In the six included studies (14–19) reporting the cases of depression in both the study group and the control group, the rates of events of depression on average in women with dysmenorrhea were 332/1317 (25.2%), while the control subjects without dysmenorrhea were 226/1833 (12.3%); the absolute effect of dysmenorrhea on depression was 89 more per 1,000 (from 54 to 123 more); the overall quality of the evidence was MODERATE.

**Table 2:** GRADE-profiler summary of evidence for the effects of primary dysmenorrhea and depression.

**Quality assessment**							**No. of patients**		**Effect**		**Quality**	**Importance**
**No. of studies**	**Design**	**Risk of bias**	**Inconsistency**	**Indirectness**	**Imprecision**	**Other considerations**	**Dysmenorrhea**	**Control**	**Relative (95%CI)**	**Absolute**		
**Depression (assessed with: the Beck Depression Inventory, the Edinburgh Depression Scale, and the PROMIS etc.)**												
6	Observational studies	Serious^a^	No serious inconsistency	Serious^b^	No serious imprecision	Very strong association^c^increased effect for RR ~1^d^^,^^e^	332/1317 (25.2%)	226/1833(12.3%)	RR 1.72 (1.44 to 2.0)	89 more per 1000 (from 54 more to 123 more)	⊕⊕⊕○ MODERATE	CRITICAL

*CI, confidence interval; RR, relative risk; PROMIS, The NIH Patient Reported Outcomes Measurement Information System. GRADE Working Group grades of evidence:*

***High quality:** Further research is very unlikely to change our confidence in the estimate of effect*.

***Moderate quality:** Further research is likely to have an important impact on our confidence in the estimate of effect and may change the estimate*.

***Low quality:** Further research is very likely to have an important impact on our confidence in the estimate of effect and is likely to change the estimate*.

***Very low quality:** We are very uncertain about the estimate*.

a *Selection bias and detection bias were observed in the 3 included studies*.

b *Some included studies suggested that other factors might also play important roles in the relationship between dysmenorrhea and depression*.

c *Very large sample, total 3,150 participants from 6 studies were included, combined RR indicated that women with dysmenorrhea were 1.72 times more likely to have depression than those women without dysmenorrhea (RR = 1.72, 95%CI: 1.44–2.0, P < 0.001)*.

d *Several included studies managed to adjust the confounding factors (i.e., age, occupational class, education, and marital status, etc.), which increased its effect of evidence*.

e *Some included studies reported that the severity of dysmenorrhea has a dose-response effect on depression*.

### Meta-Analysis

**Figure 2:**
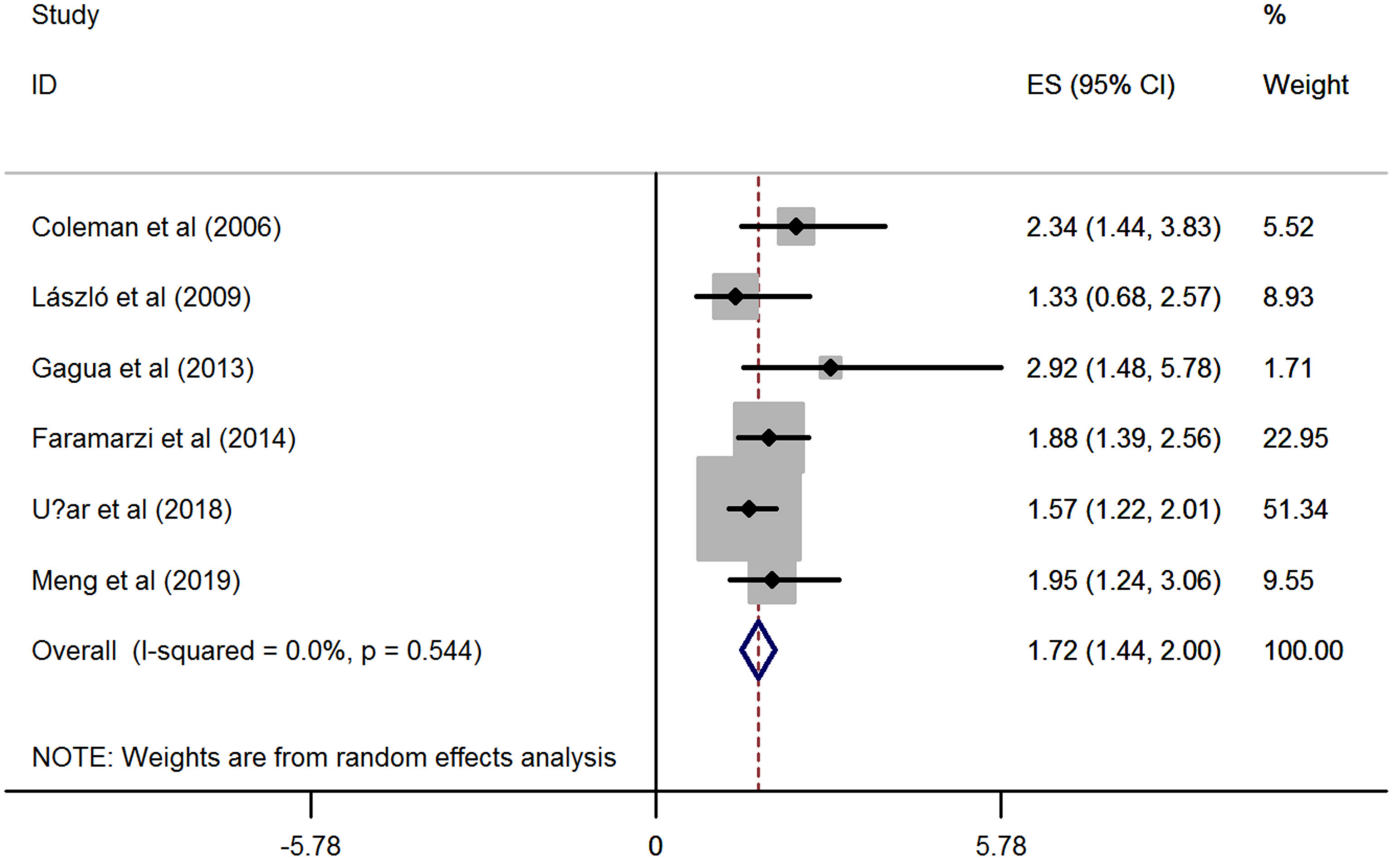
Forest plots of meta-analysis based on six eligible studies providing the cases of depression.

**Figure 3:**
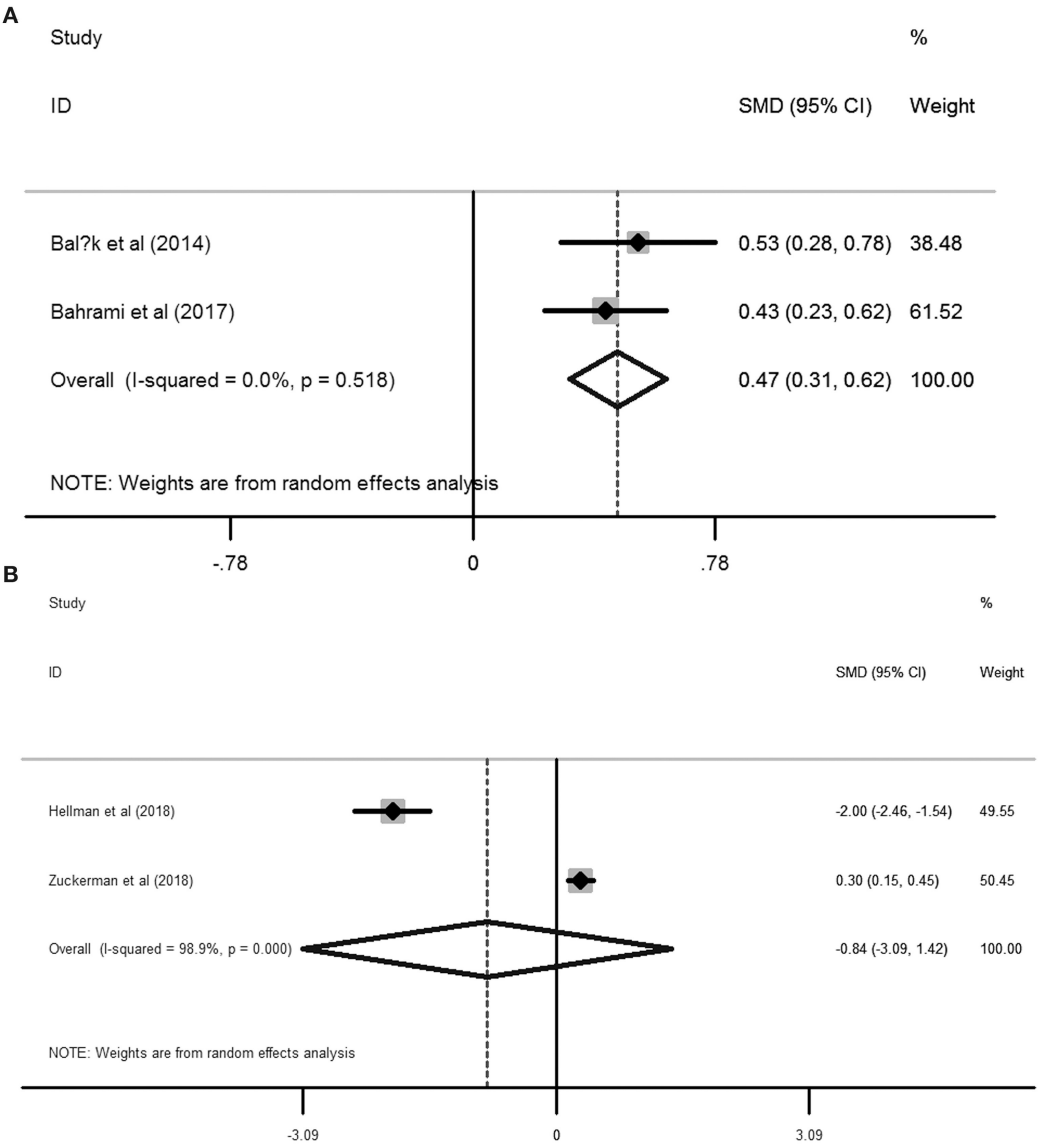
Forest plots of meta-analysis based on the two studies reporting the BDI scores **(A)** and the two studies reporting the PROMIS T-Score **(B)**.

Based on the six included studies (14–19) providing the depression cases in women with dysmenorrhea and without dysmenorrhea, the pooled RR indicated that dysmenorrhea had a significantly higher risk of depression compared to non-dysmenorrhea (*RR* = 1.72, 95%CI: 1.44–2.0, *P* < 0.001; heterogeneity: *I*^2^ = 0%, *P* = 0.544; Figure 2). In line with this finding, synthesis results from two studies (20, 21) provided the BDI scores also suggested that women complained with dysmenorrhea had significantly higher values in BDI scores as compared with the control group without dysmenorrhea (*SMD* = 0.47, 95%CI: 0.31–0.62, *P* < 0.001; heterogeneity: *I*^2^ = 0%, *P* = 0.518; Figure 3A). However, when focused on the two eligible studies (22, 23) providing the PROMIS T-Score, the combined effect demonstrated that there was no significant difference between women with dysmenorrhea and those without dysmenorrhea (*SMD* = −0.84, 95%CI: −3.09–1.42, *P* = 0.466) and substantial heterogeneity had been identified (*I*^2^ = 98.9%, *P* < 0.001; Figure 3B).

The above meta-analyses revealed that women with dysmenorrhea were more likely to encounter depressive disorder than those without dysmenorrhea. Nevertheless, pooled SMD from the two included studies (22, 23) reporting the PROMIS T-Score failed to present a positive association between dysmenorrhea and depression. Between the 2 studies, Zuckerman et al. (23) confirmed that dysmenorrhea was associated with a higher risk of depression than those without dysmenorrhea (PROMIS T-Score: 53.6 ± 9.2 vs. 50.9 ± 8.8), while in another study developed by Hellman et al. (22) indicated that dysmenorrhea was associated with a lower risk of depression (PROMIS T-Score: 53 ±1 vs. 55 ± 1). Therefore, substantial heterogeneity has been arisen due to these contradictory results.

### Sensitivity Analysis

To assess the influence of a single study on the overall RR, we have further conducted the sensitivity analyses based on the six included studies (14–19) providing the depression cases. As shown in Supplementary Figure 2 and Supplementary Table 3, sensitivity analyses revealed that there was no substantial change in the new overall pooled RR after omitting any of the six studies. The newly generated RR ranged from 1.67 (95% CI: 1.35 to 1.99, *P* < 0.001) to 1.88 (95% CI: 1.48 to 2.29, *P* <0.001). On the other hand, the heterogeneity also yielded similar results, all of the *I*^2^ were 0.0%. These results demonstrated that no single study dominated the overall synthetic RR on the association between dysmenorrhea and depression, in which the pooled effect estimate in this meta-analysis was robust.

### Publication Bias

**Figure 4:**
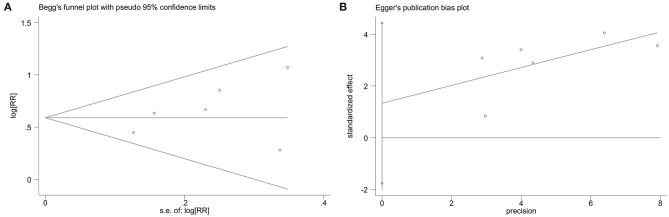
Begg's **(A)** and Egger's **(B)** tests to detect publication bias.

The funnel plots, Begg's rank correlation test, and Egger's linear regression revealed that there was no significant publication bias among the included studies (Begg's, *P* > |z| = 0.133; Egger, *P* > |t| = 0.298, 95%CI: −1.76–4.44) (Figure 4).

## Discussion

Primary dysmenorrhea or menstrual pain, as characterized by painful menstrual cramps without any discernable macroscopic pelvic pathology, is the most prevalent gynecological disorder in those of child-bearing age. Despite having a high prevalence and worsening the quality of life, dysmenorrhea is disregarded in the pain community. In essence, dysmenorrhea could be classified as a genuine chronic pain condition and accompanied by headache, vomiting, nausea, and fatigue. Moreover, dysmenorrhea can affect the social life as well as the mental health of an individual. It is known that there is a close association between pain and depression (24). Since the first publication (25) reporting the relationship between dysmenorrhea and depressive symptoms, by Bloom et al. in 1978, there is mounting evidence indicates that dysmenorrhea patients are at high risk of depression (7, 10–14). However, we should also note that several studies (13, 14) failed to find a positive association between dysmenorrhea and depressive disorder.

The present meta-analysis revealed that women with primary dysmenorrhea conferred a 1.72-fold increased risk of depressive symptoms when compared to the healthy general population without dysmenorrhea (pooled *RR* = 1.72, 95%CI: 1.44–2.0, *P* < 0.001). Besides, we also found that women with dysmenorrhea had significantly higher values in BDI scores than non-dysmenorrhea individuals (*SMD* = 0.47, 95%CI: 0.31–0.62, *P* < 0.001). The average rates of depression events were 332/1,317 (25.2%) in women with dysmenorrhea and 226/1,833 (12.3%) in those healthy individuals without dysmenorrhea. The quality of the evidence of the present meta-analysis was regarded to MODERATE. In addition, some other features of the included studies also indicated our study was robust, including high methodological quality, no substantial heterogeneities, stable sensitivity analysis, and no significant publication bias of the eligible studies.

Though the present meta-analysis suggested that primary dysmenorrhea elevated the risk of depressive symptoms, the clear-cut etiology for such association has not yet been fully established (14, 15). Progressed researches indicate multifactorial mechanisms may contribute to the high prevalence of depressive disorder in women with primary dysmenorrhea, including recurrent and chronic menstrual pain (19), hormone-responsive disorders (20), increased pro-inflammatory cytokines (14, 16), and poor school or social performances (20, 21). These factors independently or collectively contribute to someone with primary dysmenorrhea are more liable to suffer from depression.

The association of depressive symptoms with dysmenorrhea could be explained as the adverse effect of chronic pain on women's mental health. Depressive disorders are known to exacerbate or co-occur with various forms of chronic pain (26). The prevalence of depression is high in subjects with chronic pain syndromes (24). Primary dysmenorrhea occurs on a regular menstrual cycle for most who menstruate, and this is considered to be a genuine chronic pain condition. Menstrual pain could be conceptualized as a stressor and exacerbate depressive symptoms (27). Depression commonly occurs in response to dysmenorrhea and can be anticipated in the next menstrual period (28).

The pathogenesis of primary dysmenorrhea is the excess secretion of uterine prostaglandins which are controlled by both progesterone and estrogen (29). Hormonal fluctuations during the menstrual cycle affect the regulation of emotions by their effects on the brain (30). Estrogen and progesterone have been implicated in depressive symptomatology in many women (31). It was suggested that variations in ovarian progesterone and estrogen level are associated with a high risk of depression (32). Both the high concentrations of estrogen and prostaglandin are probable mechanisms of dysmenorrhea (11)^.^ Estrogen leads to mood disorders via modulating the expression of genes that code for tryptophan hydroxylase and the serotonin transporter (33). Lokuge et al. (34) demonstrated that the variations of estrogen-related depression in women might be correlated to the regulation of the serotonin pathway, causing an alteration in serotonin neurotransmission. Progesterone, another key gonadal hormone for controlling the production of prostaglandin, causes dysfunctional mood regulation by regulating neurotransmitter synthesis, release, and transport (35). In addition to prostaglandins, estrogen, and progesterone, other chemicals such as vasopressin and phospholipids released during dysmenorrhea might also be associated with depressive disorder (36). Furthermore, aberrant circulating cortisol levels were associated with pain sensitivity in women with dysmenorrhea, which made them more liable to depression (37). For other physiological mechanisms, excessive and prolonged activation of the hypothalamic-pituitary-adrenal axis resulting in impaired follicular development might be one of the underlying pathogenesis between stress and dysmenorrhea (38). And there were numerous studies have confirmed the relationship between stress and depression (39). Therefore, the high prevalence of depressive symptoms in women with primary dysmenorrhea might be partially caused by high stress.

In addition to the effects of the fluctuating hormones, previous studies have revealed that depression was associated with the increased level of several pro-inflammatory cytokines (i.e., tumor necrosis factor α and interleukins) which was considered leading a deficit in serotonin and melatonin via the kynurenine pathway (40–42). Intriguingly, a case-control study (43) demonstrated that many pro-inflammatory cytokines were up-regulated in peripheral blood mononuclear cells in primary dysmenorrhoeic young women during the menstrual period.

Primary dysmenorrhea dramatically affects one's social life or school performance, which might also play a critical role in the association between primary dysmenorrhea and depression. Dysmenorrhea is one of the main causes of school and work absences among young women, with 14% to 52% reporting absenteeism (44). Those who complained about recurrent dysmenorrhea tended to have increased levels of serious stress in daily living and decreased productivity, creativity, and job performance, which were considered as the driving factors for depression (8, 45). The depressive disorder occurs in both sexes, but during puberty, girls are at three times higher risk than boys due to the great personal changes since that time (46). Ge et al. (47) also demonstrated that entry into adolescence is a period of elevated vulnerability to depression for girls.

Since a positive association between dysmenorrhea and depressive disorder, interventions to alleviate dysmenorrhea or depression may have a great impact on the quality of life for the sufferers. Several pharmacological interventions have been reported for the management of dysmenorrhea, including non-steroid anti-inflammatory drugs (NSAIDs), combined oral contraceptives, Danazol, and Leuprolide acetate (48, 49), etc. However, potential adverse events caused by these drugs should be noted. NSAIDs may cause gastrointestinal discomfort and hemorrhage. Hormone therapy may cause irregular uterine bleeding. In addition to the drugs used for dysmenorrhea, psychotropic medications for depression therapies may also play roles to wear off depression. However, long-term use of these drugs may cause drug dependency. As aforementioned, dysmenorrhea adversely affects mood and consequently affects the sufferer's attitude and relationships with family and peers, including poor concentration and an inability to participate in social activities. Therefore, more social supports may be a way to decrease the serious stress of the dysmenorrhea sufferers in daily living. Besides, it has been assumed that behavioral interventions, i.e., relaxation training, biofeedback, and mind-body awareness, may positively help to alleviate dysmenorrhea and thus decrease the risk of depression (50). However, these interventions need to be viewed with caution due to the data supporting these treatments are still limited. Therefore, more RCTs with large sample sizes are warranted to be done in this area.

To our knowledge, this is the first study for quantifying the association between primary dysmenorrhea and the risk of depression by conducting a meta-analysis. However, several inherent limitations were also identified in this study. On one hand, all the included studies were observational designed, either cross-sectional or case-control trials, which indicated that the direction of causality between dysmenorrhea and risk of depression was not so clear. On the other hand, though both the pooled RR for the six studies reporting the cases and the pooled SMD from the two included studies reporting BDI scores have confirmed the positive association between primary dysmenorrhea and depression, the synthetic SMD from the two included studies providing the PROMIS T-Score did not support women with dysmenorrhea have a significantly higher score than the healthy controls (*SMD* = −0.84, 95%CI: −3.09–1.42, *P* = 0.466, *I*^2^ = 98.9%, *P* < 0.001). This inconsistency might cause by the limited studies (two eligible studies) were included in this analysis. In line with the combined RR and the combined SMD derived from BDI scores, Zuckerman et al. (23) also observed a positive association between dysmenorrhea and higher risk of depression (*SMD* = 0.30, 95%CI: 0.15–0.45), while Hellman et al. (22) demonstrated that dysmenorrhea was correlated with a lower risk of depression (*SMD* = −2.00, 95%CI: −2.46 to −1.54; Figure 3B). This contradictory outcome contributes to a non-significant relationship between dysmenorrhea and depression (*SMD* = −0.84, 95%CI: −3.09–1.42, *P* = 0.466) as well as a substantial heterogeneity (*I*^2^ = 98.9%, *P* < 0.001; Figure 3B) when pooling these two studies. Unfortunately, the main topic of both Zuckerman et al. and Hellman et al.'s studies was not focused on the association between dysmenorrhea and depression, the authors just mentioned the PROMIS T-Score between the healthy controls and the dysmenorrhea sufferers and no further discussions related to dysmenorrhea and depression were performed in their studies. As a result, additional well-designed prospective cohorts with a large sample are still warranted to validate the evidence of the high risk of depressive disorder in women with primary dysmenorrhea. Last, we should also note that different validation tools were employed for assessing either primary dysmenorrhea or depression in the 10 included studies, which might affect the outcomes among different studies. In addition to different validation measurements, other characteristics of the included studies are also different, such as diverse study design, sample size, age of the participants, countries, age at menarche, menstrual bleeding duration, and comorbidities. Therefore, we should be cautious when interpreting our results in clinical practice because of these confounding factors.

In conclusion, the present systematic review and meta-analysis suggest that women with primary dysmenorrhea have a significant higher prevalence of depression than those without dysmenorrhea. Understanding such a potential relationship is important in increasing awareness of assessment for the depressive symptoms, improving the quality of life, and providing better-quality care and proper intervention for women with dysmenorrhea.

## Data Availability Statement

The original contributions presented in the study are included in the article/Supplementary Material, further inquiries can be directed to the corresponding author/s.

## Author Contributions

SZ project development and data collection. WW data collection and conceptualization. RK methodology and investigation. XW and SZ original draft and methodology. All authors contributed to the article and approved the submitted version.

## Conflict of Interest

The authors declare that the research was conducted in the absence of any commercial or financial relationships that could be construed as a potential conflict of interest.

## Publisher's Note

All claims expressed in this article are solely those of the authors and do not necessarily represent those of their affiliated organizations, or those of the publisher, the editors and the reviewers. Any product that may be evaluated in this article, or claim that may be made by its manufacturer, is not guaranteed or endorsed by the publisher.

## Supplementary Material

Supplementary Figure 1 PRISMA checklist.

Supplementary Figure 2 Sensitivity analysis after each study was excluded by turns.

Supplementary Table 1 The methodological quality of the cross-sectional studies.

Supplementary Table 2 Newcastle-Ottawa Scale (NOS) assessment of the quality of the case-control studies.

Supplementary Table 3 Sensitivity analysis after each study was excluded by turns.
