# Supplementary figures and images for: Significant Increase in Depression in Women With Primary Dysmenorrhea: A Systematic Review and Cumulative Analysis
Source: Front Psychiatry. 2021 Aug 5;12:686514. doi: 10.3389/fpsyt.2021.686514 (PMC8374105; doi:10.3389/fpsyt.2021.686514)

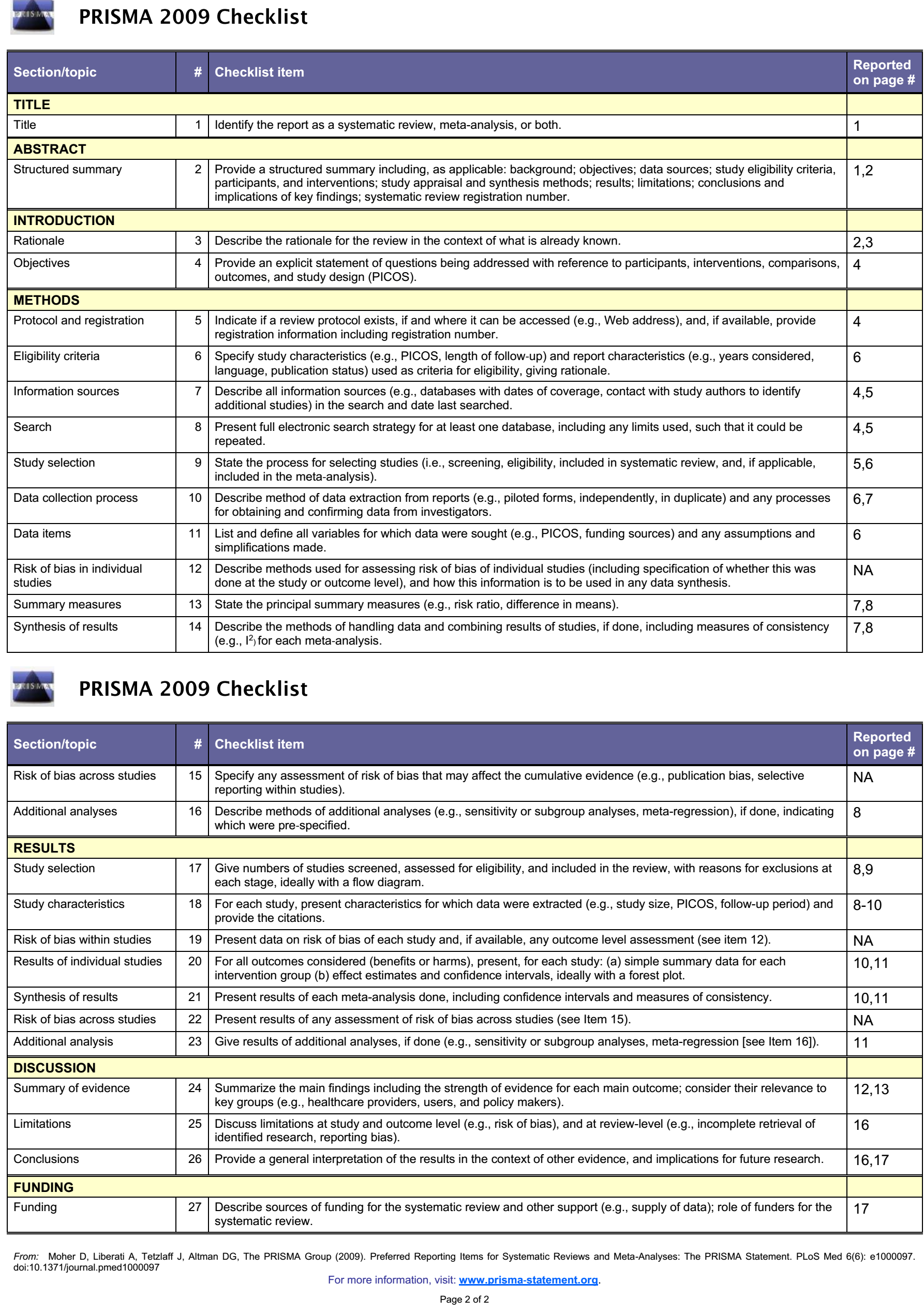

Supplement: Supplementary Figure 1 — PRISMA checklist. [file Image_1.TIF]

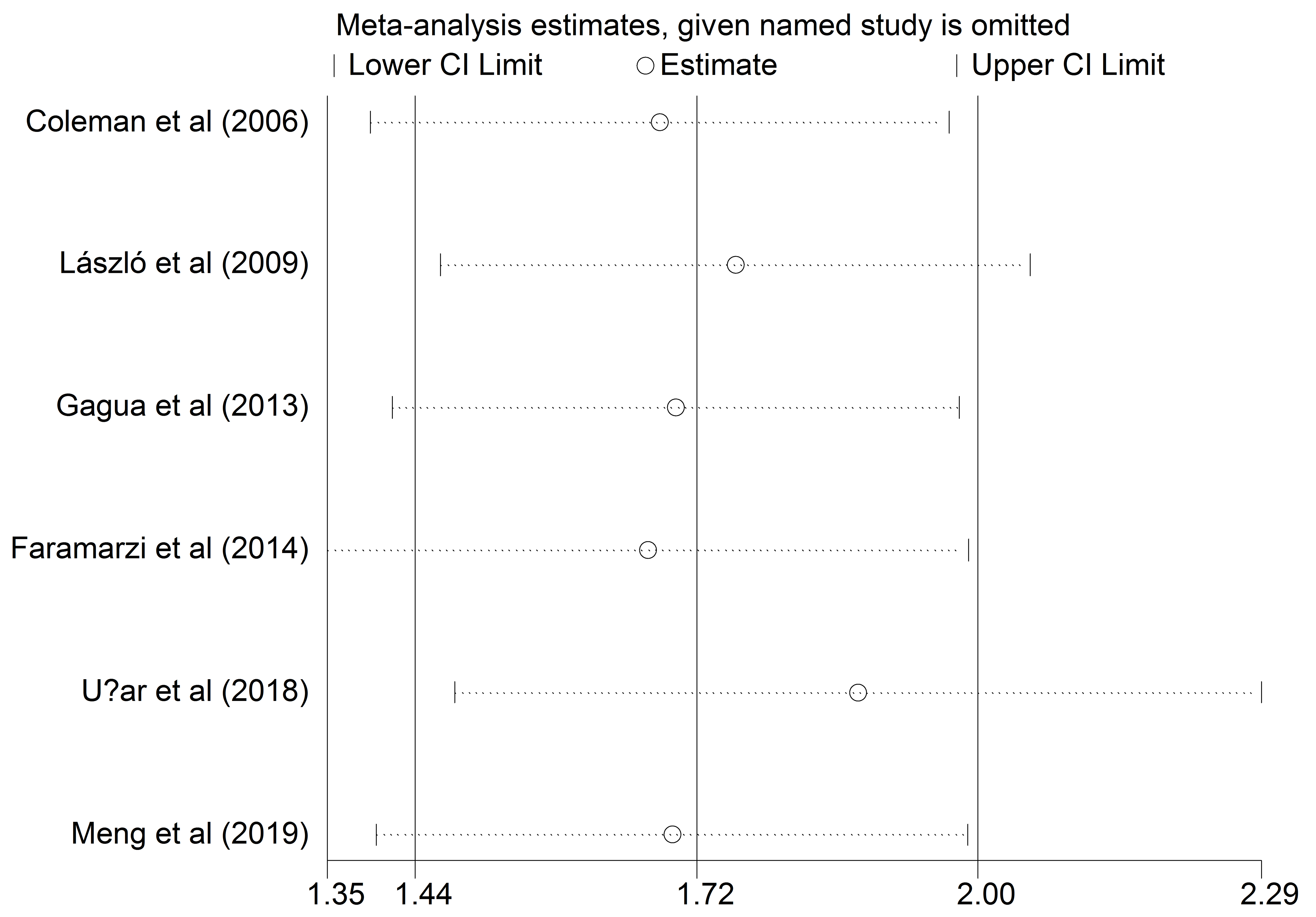

Supplement: Supplementary Figure 2 — Sensitivity analysis after each study was excluded by turns. [file Image_2.TIF]
