# Supplementary material for: Significant Increase in Depression in Women With Primary Dysmenorrhea: A Systematic Review and Cumulative Analysis
Source: Front Psychiatry. 2021 Aug 5;12:686514. doi: 10.3389/fpsyt.2021.686514 (PMC8374105; doi:10.3389/fpsyt.2021.686514)
Supplement: Supplementary Table 1 — The methodological quality of the cross-sectional studies. [file Table_1.doc]

Supplementary Table 1. The methodological quality of the cross-sectional studies.

| Item | Coleman [15] (2006) | Faramarzi  [17] (2014) | Bahrami [21] (2017) | Hellman [22] (2018) | Zuckerman  [23] (2018) |
| --- | --- | --- | --- | --- | --- |
| 1) Define the source of information (survey, record review) | Yes | Yes | Yes | Yes | Yes |
| 2) List inclusion and exclusion criteria for exposed and unexposed subjects (cases and controls) or refer to previous publications | Yes | Yes | Yes | Yes | Yes |
| 3) Indicate time period used for identifying patients | Yes | Yes | Yes | Yes | Yes |
| 4) Indicate whether or not subjects were consecutive if not population-based | Yes | Yes | Yes | Yes | Yes |
| 5) Indicate if evaluators of subjective components of study were masked to other aspects of the status of the participants | NO | NO | Yes | NO | NO |
| 6) Describe any assessments undertaken for quality assurance purposes (e.g., test/retest of primary outcome measurements) | Yes | Yes | NO | NO | NO |
| 7) Explain any patient exclusions from analysis | NO | Yes | Yes | NO | Yes |
| 8) Describe how confounding was assessed and/or controlled. | Yes | NO | Yes | Yes | Yes |
| 9) If applicable, explain how missing data were handled in the analysis | NO | Yes | Yes | NO | Yes |
| 10) Summarize patient response rates and completeness of data collection | Yes | NO | NO | Yes | NO |
| 11) Clarify what follow-up, if any, was expected and the percentage of patients for which incomplete data or follow-up was obtained | NO | NO | NO | NO | NO |
