# Supplementary material for: Significant Increase in Depression in Women With Primary Dysmenorrhea: A Systematic Review and Cumulative Analysis
Source: Front Psychiatry. 2021 Aug 5;12:686514. doi: 10.3389/fpsyt.2021.686514 (PMC8374105; doi:10.3389/fpsyt.2021.686514)
Supplement: Supplementary Table 2 — Newcastle-Ottawa Scale (NOS) assessment of the quality of the case-control studies. [file Table_2.doc]

| Study | Selection | | | | Comparability | | Exposure/Outcome | | | Total scores |
| --- | --- | --- | --- | --- | --- | --- | --- | --- | --- | --- |
|  | 1 | 2 | 3 | 4 | 5 | 6 | 7 | 8 | 9 |  |
| László et al (14), 2009 | Yes | Yes | Yes | Yes | No | No | Yes | No | No | 5 |
| Gagua et al (16), 2013 | Yes | Yes | Yes | Yes | No | No | Yes | No | No | 5 |
| Balık et al (20), 2014 | Yes | Yes | Yes | Yes | Yes | No | Yes | No | No | 6 |
| Uçar et al (18), 2018 | Yes | Yes | Yes | Yes | No | Yes | Yes | Yes | No | 7 |
| Meng et al (19), 2019 | No | Yes | Yes | Yes | Yes | No | Yes | No | No | 5 |

Supplementary Table 2. Newcastle-Ottawa Scale (NOS) assessment of the quality of case-control studies.

NOTE: 1. indicates that the exposed cohort was representative of the population; 2. Indicates that the non-exposed cohort was drawn from the same population; 3. Indicates that the exposure ascertainment was from secure records or a structured interview; 4. Indicates that outcome of interest was not present at start of study; 5. Indicates that the cohorts were comparable for age and sex; 6. Indicates that the cohorts were comparable on all additional factor(s) reported; 7. Indicates that outcome was assessed from a secure record; 8. Indicates that follow-up was long enough for outcomes to occur; 9. Indicates that follow-up was complete.
