# Supplementary material for: Significant Increase in Depression in Women With Primary Dysmenorrhea: A Systematic Review and Cumulative Analysis
Source: Front Psychiatry. 2021 Aug 5;12:686514. doi: 10.3389/fpsyt.2021.686514 (PMC8374105; doi:10.3389/fpsyt.2021.686514)
Supplement: Supplementary Table 3 — Sensitivity analysis after each study was excluded by turns. [file Table_3.doc]

Supplementary Table 3. Sensitivity analysis after each study was excluded by turns.

| Study omitted | RR (95% CI) for remainders | Heterogeneity | |
| --- | --- | --- | --- |
|  |  | *I*^2^ *P* | |
| *The 6 included studies that provided the sufficient data which could be calculated for the pooled RR with 95% CI:* | | | |
| Coleman et al., (2006) (15) | 1.69 (1.40, 1.97), *P*<0.001 | 0.0% | 0.568 |
| László et al., (2009) (14) | 1.76 (1.47, 2.06), *P*<0.001 | 0.0% | 0.510 |
| Gagua et al., (2013) (16) | 1.70 (1.42, 1.98), *P*<0.001 | 0.0% | 0.588 |
| Faramarzi et al., (2014) (17) | 1.67 (1.35, 1.99), *P*<0.001 | 0.0% | 0.454 |
| Uçar et al., (2018) (18) | 1.88 (1.48, 2.29), *P*<0.001 | 0.0% | 0.586 |
| Meng et al., (2019) (19) | 1.70 (1.40, 1.99), *P*<0.001 | 0.0% | 0.438 |

Abbreviation: RR=relative risk; CI=confidence interval.
